# Supplementary material for: The Marine Seagrass Halophila stipulacea as a Source of Bioactive Metabolites against Obesity and Biofouling
Source: Mar Drugs. 2020 Jan 29;18(2):88. doi: 10.3390/md18020088 (PMC7074557; doi:10.3390/md18020088)
Supplement: Supplementary file 1 [file marinedrugs-18-00088-s001.pdf]

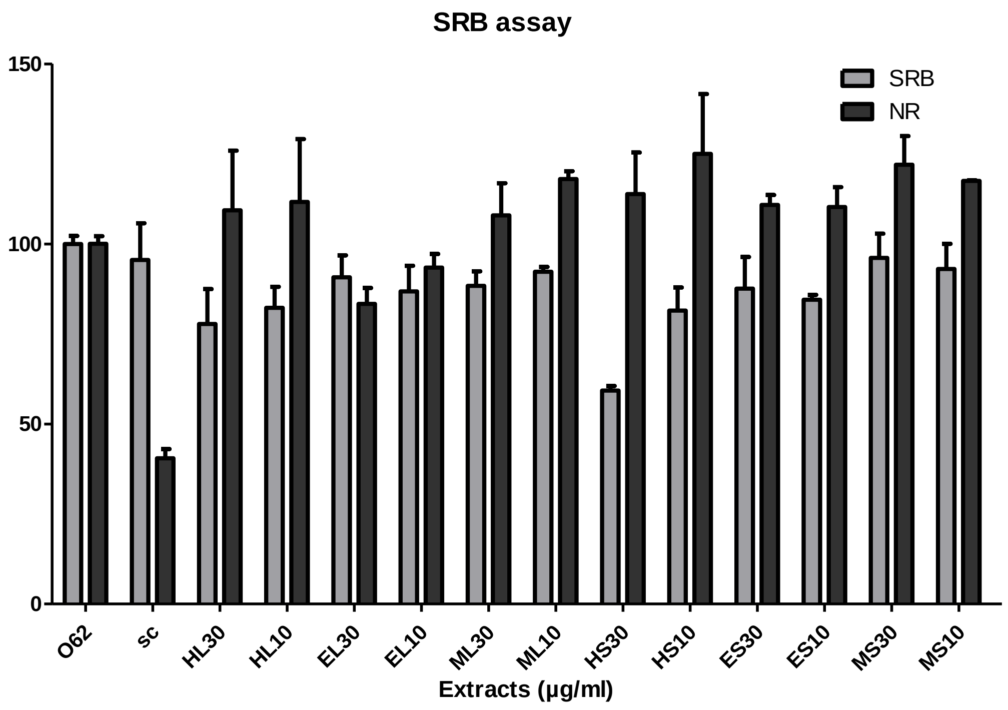

Supplementary Figure S1. Antisteatosis assay of different extracts (30 μg/ml, 10 μg/ml) in fattened liver cells (HepG2 cell line). Nile red (NR) fluorescence indicating neutral lipid content of cells; sulforhodamine B (SRB), viability test. Cells were overloaded with sodium oleate at 62 μM.

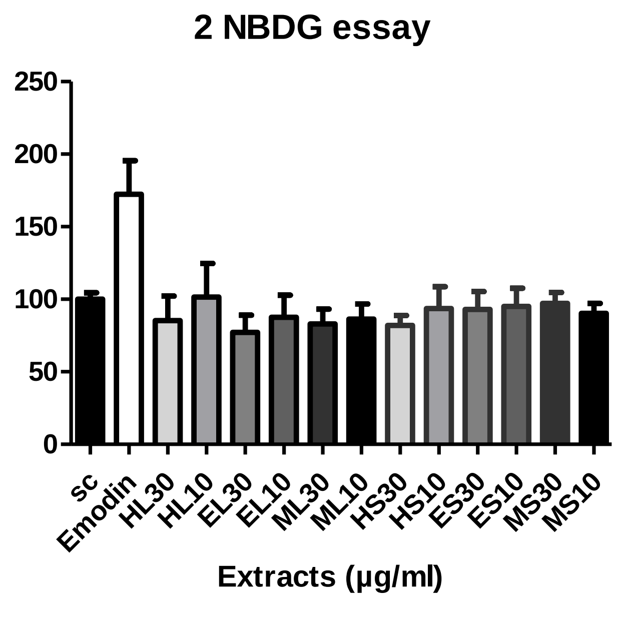

Supplementary Figure S2. Glucose uptake assay of different extracts (30 μg/ml, 10 μg/ml) using 2NBDG incorporation in HepG2 cells.

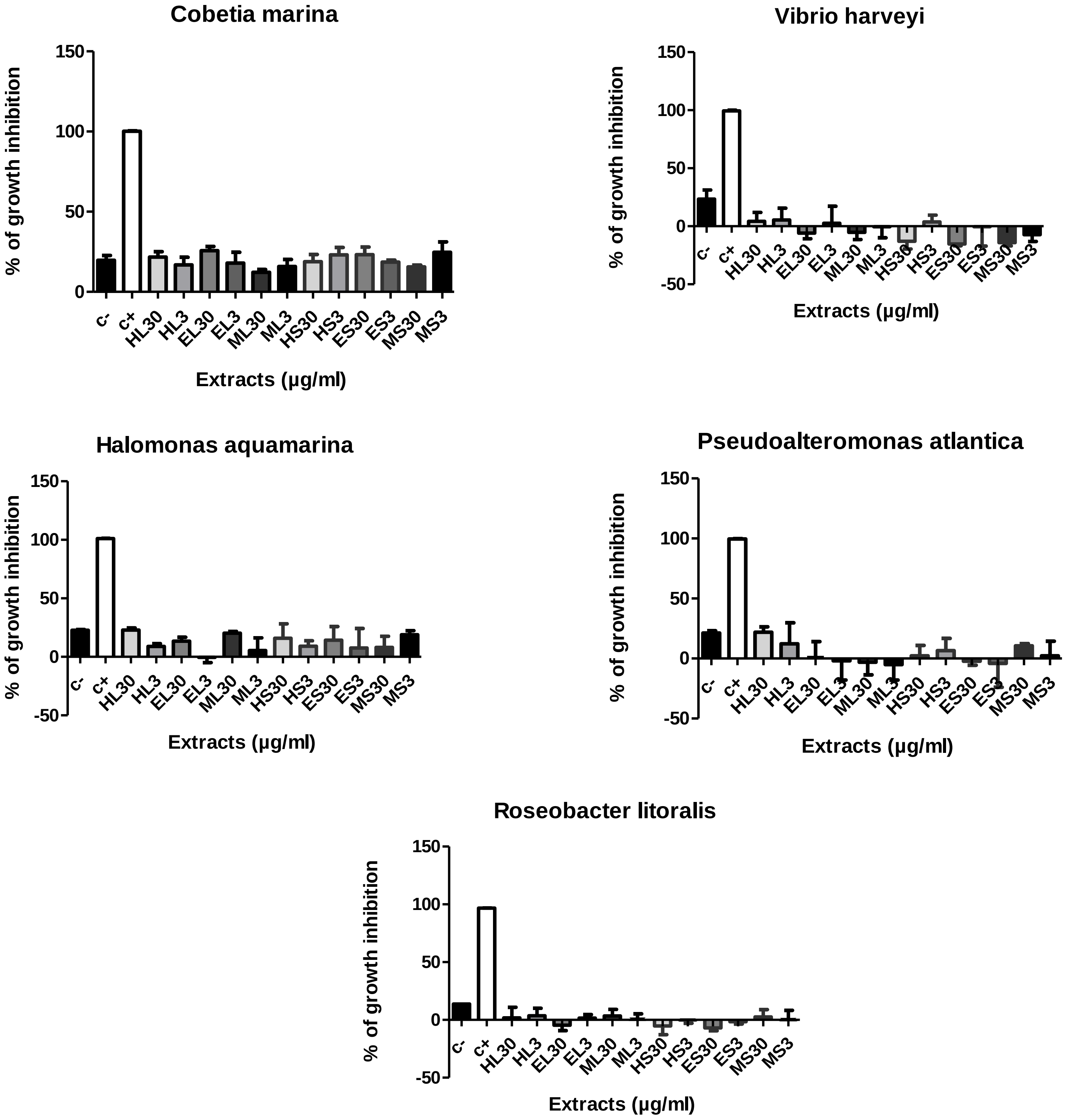

Supplementary Figure S3. Bioassay with marine fouling bacteria using different extracts at 3 µg/ml and 30 µg/ml.

Supplementary Table S1. Identified compounds from molecular networking with GNPS, based on LC-MS/MS data (positive mode) obtained from different extracts (HL, EL, ML, HS, ES, MS). A color code highlights some compounds with known function related to the studied bioactivities (yellow: cytotoxicity; blue: obesity; green: biofouling).

Class	Compound Name	m/z	HL	EL	ML	HS	ES	MS
**Polyphenols**	Luteolin	287.055					x	x
	Spiraeoside	463.124			x			x
	Aloenin	433.113			x			x
	6''-O-Acetylgenistin	474.905	x		x	x	x	
	luteolin 4'-O-glucoside	447.129	x	x	x			x
	Diosmetin	301.071	x	x			x	x
	1,3,6-trihydroxy-2-(3-methylbut-2-enyl)xanthen-9-one	350.371	x	x	x		x	x
	2-Hyroxybiphenyl	169.08	x	x	x	x	x	x
	Isocitrate	173.043	x	x	x	x	x	x
	Esculetin	177.059	x	x	x	x	x	x
	alpha.-Cyano-4-hydroxycinnamic acid	189.052	x	x	x	x	x	x
	3-Hydroxy-4-methoxycinnamic acid	194.127	x	x	x	x	x	x
	5-Oxohexanoic Acid	128.143	x	x	x	x	x	x
	p-Hydroxyphenyllactic acid	180.154	x	x	x	x	x	x
	Dihydroartemisinin	231.116	x	x	x	x	x	x
**Terpenoids**	ginkgolide B	464.858	x	x	x	x		x
	Perillyl alcohol	155.108		x			x	
	Xanthomonic Acid	467.348	x	x	x	x	x	x
**Chlorophylls**	Pheophorbide A	593.276		x			x	
	Pheophytin	871.573			x		x	x
**Alkaloids**	3-ethyl-2,3,6,7,8,8a-hexahydropyrrolo [1,2-a]pyrazine-1,4-dione	183.078	x	x	x	x	x	x
**Vitamins**	Cholesta-5,8(9)-dien-3.beta.-ol	387.322			x			x
	Choline	104.068	x	x	x		x	x
**Fatty Acid**	1-Arachidonoylglycerol	394.353		x				x
	2,4-Diaminobutyric acid	118.086	x			x		
	9-Oxo-10E,12Z-octadecadienoic acid	294.183			x		x	x
	13-Keto-9Z,11E-octadecadienoic acid	297.172			x	x		x
	cis-7,10,13,16-Docosatetraenoic acid	218.725		x	x		x	x
	9-Oxo-10E,12Z-octadecadienoic acid	294.181	x	x		x	x	
	13-Docosenamide, (Z)	338.342		x	x	x		x
	1-Hexadecanoyl-sn-glycerol	332.331	x	x	x	x	x	x
	11,15-Dioxo-9S-hydroxy-5Z-prostenoic acid	352.306	x	x	x	x	x	x
	Palmitoylcarnitine	399.277	x	x	x	x	x	x
	Decanedioic acid, bis(2-ethylhexyl) ester	427.378	x	x	x	x	x	x
**Carbohydrates**	D-Mannosamine	179.118	x			x		
	D-(-)-Tagatose	198.097	x	x			x	x
	Sucrose	360.15			x	x	x	x
	Uridine	114.091	x	x	x	x	x	x
	Maltulose	364.28						
**Amino acids and peptides**	L-Arginine	176.013		x			x	x
	S-Adenosyl-L-methionine	226.951		x			x	x
	L-Ornithine	116.071	x				x	x
	L-tryptophan derivative	203.034		x	x		x	x
	Lys-Leu	244.153		x	x		x	x

	4-Hydroxy-2-quinolinecarboxylic acid	190.002		x	x		x	x
	L-2,4-Diamino-4-oxobutanoate	132.991	x	x	x	x	x	
	N-Methyl-L-Glutamate	159.969	x	x	x		x	x
	Histamine	108.81	x	x		x	x	x
	Glu-Glu	277.104	x	x	x	x	x	x
	4-Quinolinecarboxylic acid	174.025		x				x
	O,S-Dimethyl acetylphosphoramidothioate	182.794	x			x		
	Dexpanthenol	206.138	x			x		
	Rolipram	274.873			x			x
	Naproxen	229.047			x			x
	N-(tert-Butoxycarbonyl)glycine	174.938		x	x		x	
	Hexaethylene glycol	283.175	x	x				x
**Synthetic compounds**	Pentamidine	339.374		x	x		x	x
	11-Deoxyprostaglandin E1	320.243	x		x	x	x	
	Cholic acid	426.322	x	x	x	x	x	
	Naltrexone	341.316	x	x	x	x	x	x
	Chloramphenicol	321.131	x	x	x	x	x	x
	Nalidixate	233.078	x	x	x	x	x	x
	Bisphenol A bis(2,3-dihydroxypropyl) ether	394.311	x	x	x	x	x	x
	Avobenzone	311.183	x	x	x	x	x	x
	Minocycline	458.348	x	x	x	x	x	x
